# Lumefantrine ameliorates DSS-induced colitis by targeting FLI-1 to suppress NF-κB signaling

**DOI:** 10.3389/fphar.2025.1614978

**Published:** 2025-07-11

**Authors:** Ji Yang, Peng Guo, Hongtao Luo, Xin Tang, Wei Liu, Xiaolin Ren

**Affiliations:** ^1^ College of Laboratory Medicine, Chengdu Medical College, Chengdu, China; ^2^ Department of Clinical Medicine, Chengdu Medical College, Chengdu, China; ^3^ Pidu District Hospital of Traditional Chinese Medicine, Chengdu, China; ^4^ School of Bioscience and Technology, Chengdu Medical College, Chengdu, China; ^5^ The Second Affiliated Hospital of Chengdu Medical College·Nuclear Industry 416 Hospital, Chengdu, China; ^6^ Key Laboratory of Target Discovery and Protein Drug Development in Major Diseases of Sichuan Higher Education Institutes, Chengdu, China

**Keywords:** Lumefantrine, Fli-1, NF-κB, inflammatory bowel disease (IBD), colitis

## Abstract

**Background:**

Current therapeutic options for inflammatory bowel disease (IBD) remain suboptimal due to limited efficacy, significant side effects, and high relapse rates, necessitating novel treatment strategies. Lumefantrine, a clinically established antimalarial drug, emerges as a compelling repurposing candidate based on its putative anti-inflammatory activity, though its efficacy and mechanism in IBD remain unexplored.

**Methods:**

A murine IBD model was induced by 3% dextran sulfate sodium (DSS). Mice received oral Lumefantrine (20 mg/kg/day) for 7 days. Disease progression was monitored via disease activity index (DAI) scoring and histological analysis. Serum cytokines (IL-1β, IL-6, TNF-α) and colonic inflammatory mediators (Cox-2, iNos) were quantified by ELISA and qPCR. Tight junction proteins (Claudin-1, ZO-1) were assessed by immunohistochemistry and Western blot. Molecular targets were identified through computational docking and pull-down assays. Additionally, NF-κB signaling modulation was assessed in lipopolysaccharide (LPS)-stimulated intestinal epithelial cells (IEC-6 and NCM460) via Western blot analysis.

**Results:**

Oral administration of Lumefantrine significantly attenuated disease activity index (DAI) scores and restored intestinal barrier integrity through upregulation of epithelial tight junction proteins Claudin-1 and ZO-1. Treated mice exhibited reduced serum levels of IL-1β, IL-6 and TNF-α, along with decreased colonic expression of inflammatory mediators cyclooxygenase-2 (Cox-2) and inducible nitric oxide synthase (iNos). Computational and experimental approaches identified FLI-1 a transcription factor upregulated in IBD colon tissues as Lumefantrine’s direct binding target. This interaction mediated suppression of NF-κB signaling, specifically downregulating phosphorylation of IκBα and p65 in LPS-stimulated intestinal epithelial cells.

**Conclusion:**

Lumefantrine ameliorates experimental colitis through FLI-1-dependent inhibition of the NF-κB pathway, demonstrating high repurposing potential as an IBD therapeutic.

## 1 Introduction

Inflammatory bowel disease (IBD), encompassing ulcerative colitis (UC) and Crohn’s disease (CD), is a chronic, immune-mediated disorder characterized by relapsing gastrointestinal inflammation (Hodson, 2016; Flynn and Eisenstein, 2019). Its complex, multifactorial etiology culminates in immune dysregulation, persistent mucosal inflammation, epithelial barrier dysfunction, and increased colorectal cancer risk (Agrawal et al., 2022; Baumgart and Carding, 2007; Terzić et al., 2010).

Central to IBD pathogenesis is the aberrant activation of the NF-κB signaling pathway, a master regulator of inflammation. Constitutive NF-κB activation in IBD tissues drives the overexpression of key pro-inflammatory cytokines (e.g., IL-1β, IL-6, TNF-α) and mediators (e.g., COX-2, iNOS), perpetuating tissue damage and inflammation (Biasi et al., 2013; Mussbacher et al., 2023; Mukherjee et al., 2024). Furthermore, this inflammatory cascade disrupts intestinal barrier integrity by downregulating tight junction proteins such as Claudin-1 and ZO-1 (zona occludens protein 1) (Cui et al., 2021).

While current therapies (aminosalicylates, immunosuppressants, glucocorticoids, biologics, and antibiotics) offer moderate efficacy (Lamb et al., 2019), the relapsing-remitting nature of IBD necessitates repeated interventions, exposing patients to significant risks of progression and treatment-related complications (Herlihy and Feakins, 2022). Consequently, the development of novel therapeutic strategies remains imperative, with targeting the NF-κB axis continuing to represent a highly promising approach.

Drug repurposing offers an efficient strategy to accelerate IBD drug discovery. Lumefantrine (chemical name: β-dibutylamine-[2,7-dichloro-9-p-chlorophenylmethyl-4-fluorene] ethanol), an FDA-approved antimalarial (Wiesner et al., 2003; Ippolito et al., 2017), emerges as a compelling candidate. Beyond its primary use, recent studies indicate Lumefantrine inhibits the transcription factor FLI-1 (friend leukemia virus integration 1), modulating processes like extracellular matrix remodeling (Rajesh et al., 2020). Critically, FLI-1 has been shown to regulate NF-κB component expression (NF-κB1/p50 and RelA/p65) (Sartori et al., 2021). These findings raise the possibility that Lumefantrine might suppress pathogenic NF-κB signaling in IBD through FLI-1 inhibition, warranting experimental verification.

Therefore, we hypothesized that Lumefantrine attenuates IBD by targeting FLI-1, thereby inhibiting NF-κB activation. This study aimed to evaluate Lumefantrine’s efficacy in a DSS-induced murine colitis model and elucidate its mechanism, focusing on FLI-1 targeting and NF-κB pathway modulation in intestinal epithelial cells.

## 2 Materials and methods

### 2.1 Chemicals and antibodies

Lumefantrine was purchased from Aladdin (Shanghai, China), Camptothecin (CPT) from Macklin (Shanghai, China), YK-4-279 from MedChemExpress (Shanghai, China), dextran sulfate sodium (DSS) from MP Biomedicals (Shanghai, China). The primary antibodies used were: Claudin-1 (Abcam, ab15098, Cambridge, United Kingdom), ZO-1 (CST, 8193P, Massachusetts, United States), p-p65 (Proteintech, 82335-1-RR, Wuhan, China), p65 (CST, 8242S, Massachusetts, United States), p-IκB (Proteintech, 82349-1-RR, Wuhan, China), IκB (Proteintech, 66418-1-Ig, Wuhan, China), β-Actin (Proteintech, 66009-1-Ig, Wuhan, China), and FLI-1 (Santa Cruz, sc-365294, Shanghai, China). HRP-conjugated secondary antibodies anti-mouse/rabbit IgG were purchased from ZSGB-Bio (Beijing, China) for IHC, and CST (Massachusetts, United States) for WB.

### 2.2 Animal model establishment

Female C57BL/6J mice were purchased from Chengdu Dossy experimental animals Co., Ltd. All procedures were approved by the Institutional Animal Care and Use Committee of Chengdu Medical College. 8-week-old (19–21 g) mice were housed under SPF conditions (22°C ± 1°C, 55% ± 5% humidity, 12-h light/dark cycle) with a 7-day acclimatization period prior to experimentation. And then 18 mice were randomly assigned to three experimental groups: (1) control group receiving standard drinking water, (2) DSS-induced colitis group administered 3% dextran sulfate sodium (DSS) in drinking water, and (3) treatment group receiving both DSS and daily oral gavage of Lumefantrine (20 mg/kg body weight) administered as a suspension in sterile distilled water. Throughout 7-day induction and treatment phase, clinical parameters - weight loss, stool consistency, and rectal bleeding - were recorded daily and quantified via standardized scoring criteria. Terminal procedures were performed on day 7 under ether anesthesia. Blood samples collected by eyeball excision were centrifuged at 3000 rpm for 10 min to isolate serum, which was subsequently stored at −80°C. Colons were excised from the ileocecal junction to the anal verge for precise length measurement, followed by systematic tissue sampling - distal colon segments were snap-frozen in liquid nitrogen for molecular analyses while middle colon sections were processed for histological evaluation.

### 2.3 Disease activity index (DAI) assessment

Disease progression was quantified using a validated Disease Activity Index (DAI) scoring system (Mukherjee et al., 2024), which evaluates three parameters: percentage weight loss, fecal bleeding severity, and stool consistency. The composite DAI score represents the arithmetic mean of these three components, with higher scores indicating more severe colitis. Detailed scoring criteria are presented in Supplementary Table S1.

### 2.4 Histopathological analysis

Mid-colon segments were fixed in 4% paraformaldehyde, paraffin-embedded, and sectioned at 5 μm thickness for hematoxylin and eosin (H&E) staining using stain kit (Solarbio, G1120, Beijing, China). Histopathological assessment was performed by a board-certified pathologist using the standardized criteria outlined in Supplementary Table S2.

### 2.5 Immunohistochemical staining (IHC)

Immunohistochemistry was performed as previously described. Briefly, antigen retrieval was conducted using citrate buffer (pH 6.0) at 95°C for 20 min. Endogenous peroxidase activity was quenched with 3% H_2_O_2_ for 15 min. Sections were incubated with primary antibodies at 4°C overnight. Sequential incubation with horseradish peroxidase (HRP)-conjugated secondary antibodies (anti-mouse/rabbit IgG; 1:200 dilution) was performed at 37°C for 30 min. Diaminobenzidine (DAB) (Beijing ZSGB-BIO, Beijing, China) chromogen was used for signal development, with hematoxylin counterstaining.

### 2.6 Inflammatory cytokine quantification (ELISA)

Serum levels of IL-1β, IL-6, and TNF-α were measured using commercial ELISA kits (Cusabio, Wuhan, China) according to the manufacturer’s protocol. Optical density was measured at 450 nm using a microplate reader (Molecular Devices, United States).

### 2.7 Quantitative real-time PCR (qRT-PCR)

Total RNA was extracted from colon tissues using TRIzol reagent (Tiangen Biotech, Beijing, China), followed by cDNA synthesis with Reverse Transcriptase (Vazyme Biotech, Nanjing, China). Primers were synthesized by Tsingke Biotech Co., Ltd. (Beijing, China), and the sequences are listed in Supplementary Table S3. Quantitative PCR amplification was conducted using the following protocol: initial denaturation at 95°C for 15 min, followed by 40 cycles of 95°C for 10 s (denaturation) and 60°C for 30 s (annealing/extension), according to the manufacturer’s instructions for SYBR Green I master mix (Roche Diagnostics, Branchburg, United States). β-*Actin* served as the endogenous control. Relative gene expression was calculated via 2^−ΔΔCt^ method.

### 2.8 Molecular docking analysis

Computational docking was performed to predict Lumefantrine-FLI-1 interactions. The FLI-1 tertiary structure was modeled using AlphaFold2. The PDB file of FLI-1 was uploaded to the PlayMolecule platform (https://playmolecule.com/) to predict potential ligand-binding pockets. Docking simulations were performed using SailVian, and binding stability was assessed through 100 ns molecular dynamics simulations.

### 2.9 Pull-down assay

Cell lysates were incubated with epoxy-activated μSphere magnetic beads (Tianyan Biotech, Wuxi, China) pre-conjugated with 20 μM Lumefantrine (4°C, 12 h rotation). After washing (3× PBS), bound proteins were eluted (0.5 mol/L NaCl) and analyzed by Western blotting. Control groups received ethanolamine-blocked beads without compound.

### 2.10 Cell culture

The Chinese Academy of Sciences Cellular Bank (Shanghai, China) provided IEC-6 and NCM460 cell lines. Both cell lines were maintained in RPMI-1640 medium supplemented with 10% fetal bovine serum (FBS), cultured in a humidified incubator (37°C, 5% CO_2_, 95% air). Cells were subcultured at 80%–90% confluence using 0.25% trypsin-EDTA.

### 2.11 Cell viability assay (CCK-8)

Cells were seeded in 96-well plates at 5,000-10,000 cells/well and allowed to adhere for 24 h. Following treatment with Lumefantrine (0–40 μM) for 24/48 h, 10 μL CCK-8 reagent (Labgic, Beijing, China) was added to each well. After 2–4 h incubation protected from light, absorbance was measured at 450 nm using a microplate reader (Molecular Devices, United States). Cell viability (%) was calculated.

### 2.12 Western blot analysis

IEC-6 was treated with 20 μg/mL LPS for 48 h, and then treated with Lumefantrine, Camptothecin (CPT) or YK-4-279 for another 24 h. Treated cells were lysed in RIPA buffer containing protease/phosphatase inhibitors (Roche, Penzberg, Germany). Lysates were centrifuged, and supernatants were quantified via BCA assay (Thermo Fisher Scientific, United States). Proteins with equal amounts (20 μL) were electrophoretic separated on 10% SDS-PAGE gels and transferred to PVDF membranes (Merck Millipore, Darmstadt, Germany). After blocking with 5% non-fat milk/TBST (1 h, RT), membranes were incubated with primary antibodies (1:1000) overnight at 4°C, followed by HRP-conjugated secondary antibodies (anti-mouse/rabbit IgG, 1:5,000) for 2 h at RT. Signals were developed using ECL Prime (Labgic, Beijing, China) and quantified via ImageJ software.

### 2.13 Statistical analysis

All experiments were repeated independently at least three times. All data are represened as mean values ±standard deviation (S.D.). GraphPad Prism 9.0 performed unpaired t-tests. Significance thresholds: *p < 0.05, **p < 0.01, ***p < 0.001, ****p < 0.0001; ns = not significant (*p* ≥ 0.05).

## 3 Result

### 3.1 Lumefantrine ameliorates DSS-Induced colitis in mice

**FIGURE 1:**
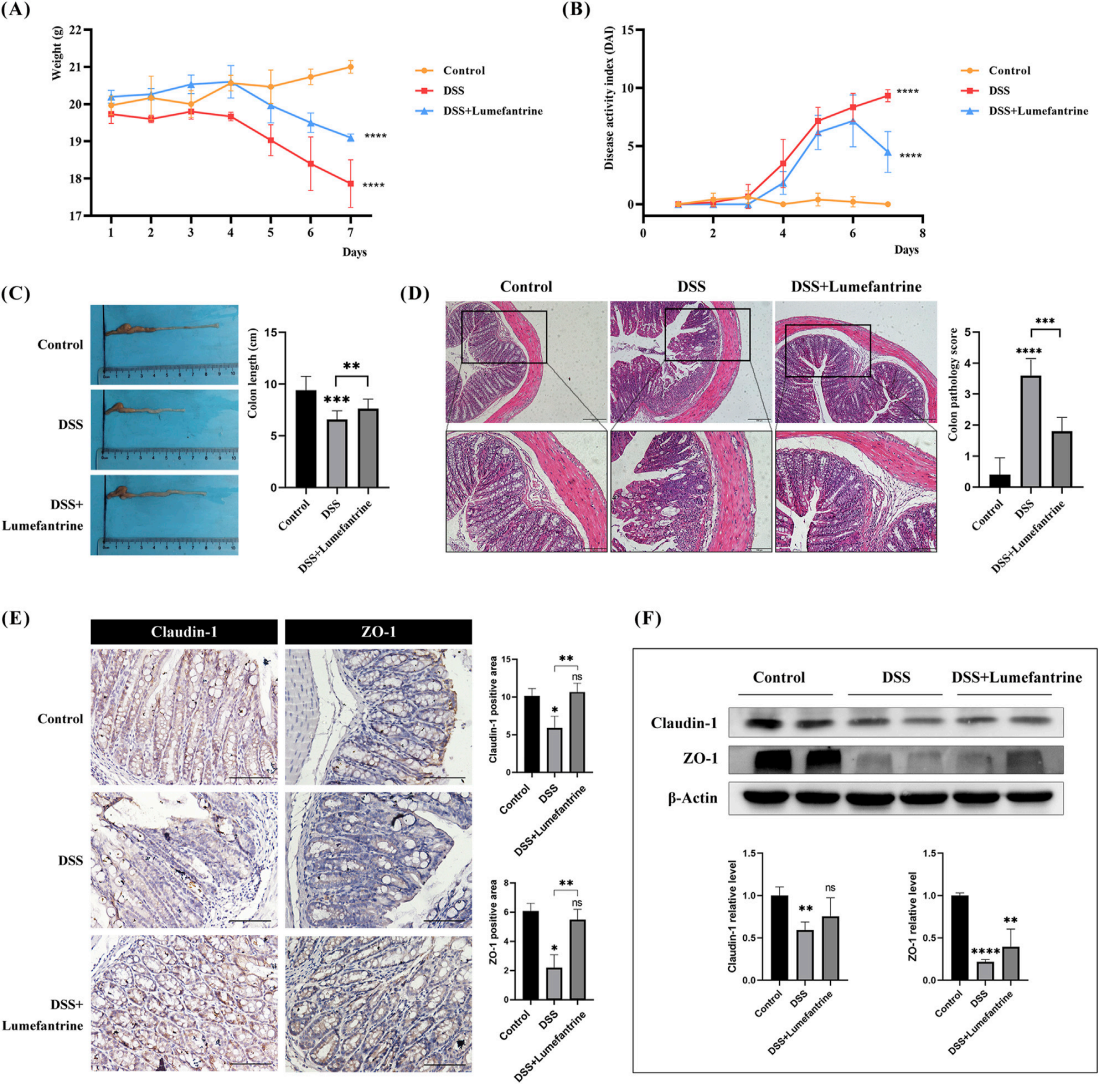
Lumefantrine ameliorates DSS-induced colitis in mice. **(A)** Body weight changes in mice. **(B)** Disease Activity Index (DAI) scores. **(C)** Colon length measurement. **(D)** Hematoxylin and eosin, (H&E)-stained colon sections (scale bar: 200 μm above and 100 μm below), showing the colon pathological score on the right side. **(E)** Immunohistochemical and **(F)** Western blot analysis of tight junction proteins Claudin-1 and ZO-1 expression in colonic tissues. (scale bar: 100 μm).

Daily monitoring revealed significant weight loss in DSS-treated mice compared to controls (Figure 1A). Lumefantrine administration markedly attenuated weight reduction. Disease activity index (DAI) scores progressively increased in model mice but were suppressed by Lumefantrine treatment (Figure 1B). Colon shortening induced by DSS was partially reversed in the treatment group (Figure 1C). Histopathological analysis demonstrated severe mucosal erosion and inflammatory infiltration in DSS mice, whereas Lumefantrine preserved epithelial continuity and reduced crypt damage (Figure 1D). Correspondingly, immunohistochemical and Western blot analyses demonstrated that DSS challenge significantly reduced colonic Claudin-1 and ZO-1 expression, indicative of impaired epithelial barrier integrity (Figures 1E,F). Lumefantrine administration restored these tight junction proteins, correlating with preserved mucosal architecture.

### 3.2 Anti-inflammatory effects of Lumefantrine

**FIGURE 2:**
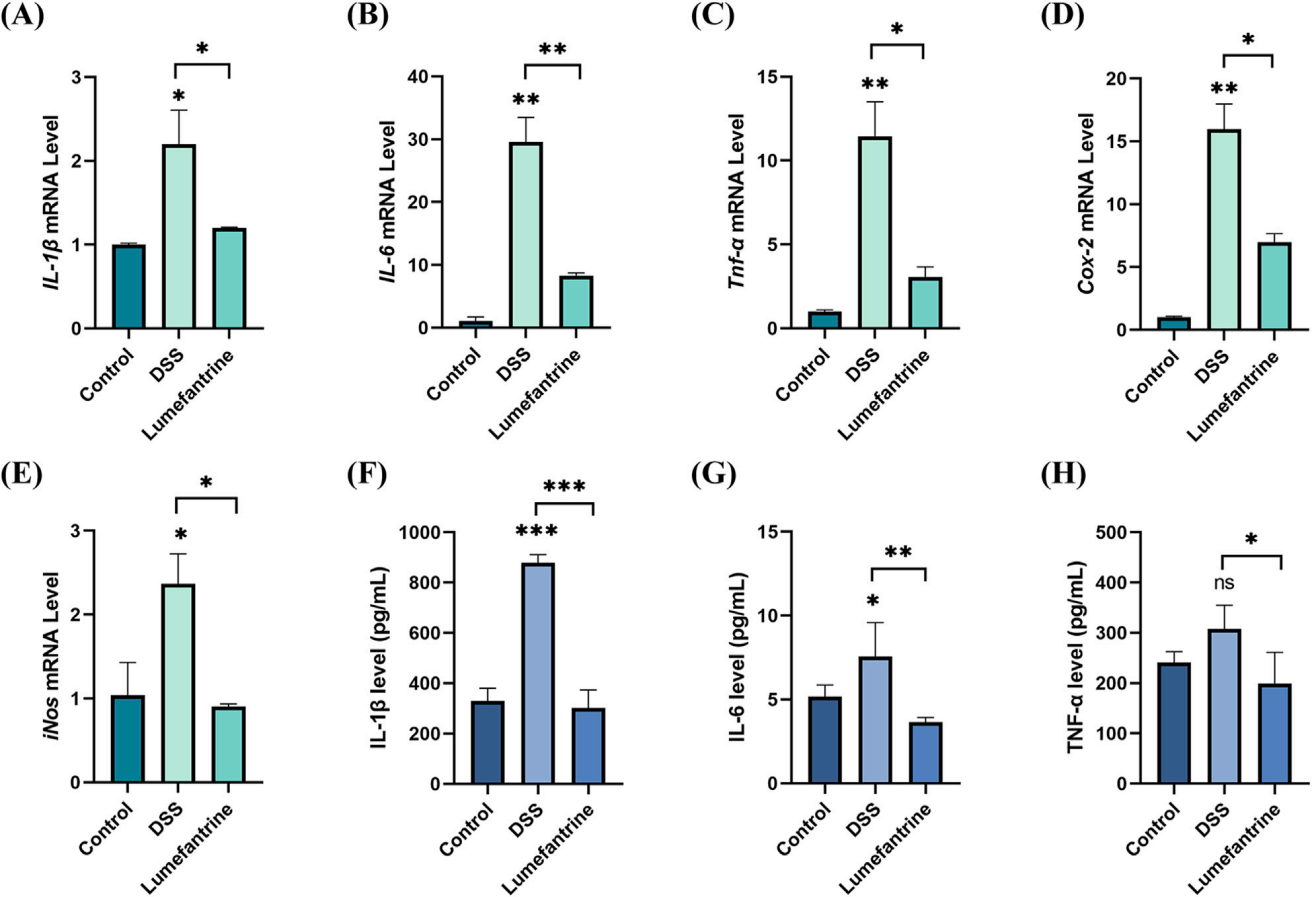
Anti-inflammatory effects of Lumefantrine. **(A–E)** mRNA expression levels of pro-inflammatory cytokines (*IL-1*β, *IL-6*, *Tnf-α*) and mediators (*Cox-2*, *iNos*) in murine model colon tissues. **(F–H)** Serum protein concentrations of IL-1β, IL-6, and TNF-α measured by ELISA.

DSS challenge significantly upregulated colonic mRNA expression of pro-inflammatory cytokines *IL-1*β, *IL-6*, *Tnf-α*, along with inflammatory mediators *Cox-2* and *iNos*. Lumefantrine administration markedly attenuated these elevations (Figures 2A–E). Serum ELISA quantification revealed corresponding decreases in protein concentrations: IL-1β, IL-6 and TNF-α all declined following Lumefantrine intervention (Figures 2F–H).

### 3.3 Computational validation of Lumefantrine-FLI-1 interaction

**FIGURE 3:**
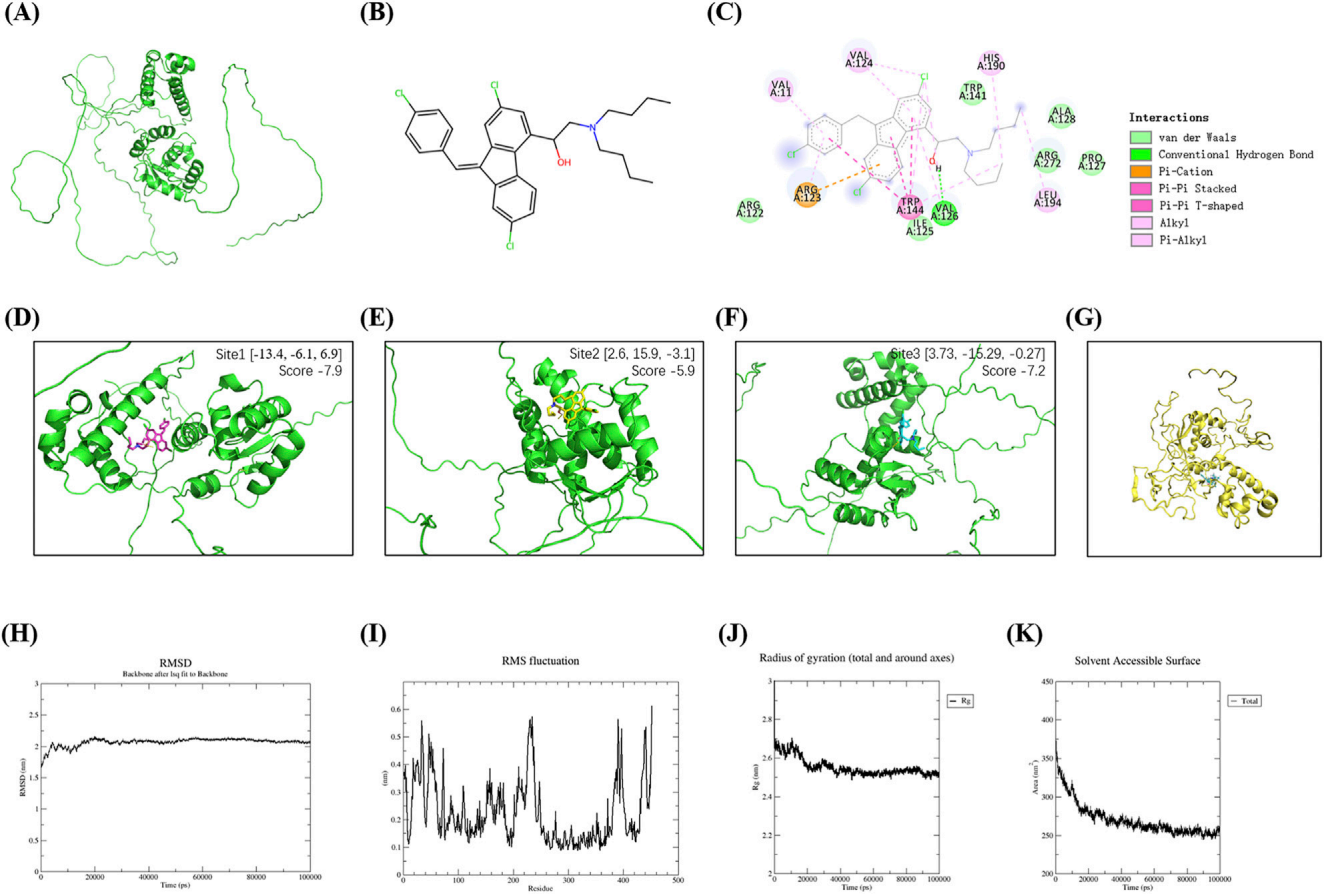
Molecular docking and dynamics simulations of Lumefantrine-FLI-1 interaction. **(A)** Predicted tertiary structure of FLI-1 (UniProt database). **(B)** Chemical structure of Lumefantrine. **(C)** 2D interaction diagram showing intermolecular force between FLI-1 and Lumefantrine. **(D–F)** Predicted ligand-binding pockets on FLI-1. **(G)** Binding conformation of the Lumefantrine-FLI-1 complex during molecular dynamics simulations. **(H–K)** Molecular dynamics parameters: Root mean square deviation [RMSD, **(H)**], root mean square fluctuation [RMSF, **(I)**], radius of gyration [Rg, **(J)**], and solvent-accessible surface area [SASA, **(K)**], confirming stable complex formation.

The three-dimensional structure of FLI-1 predicted via the UniProt database (Figure 3A) and Lumefantrine (Figure 3B) were subjected to molecular docking. The 2D interaction diagram (Figure 3C) revealed that Lumefantrine binds to FLI-1 through hydrogen bonds and van der Waals forces, with the strongest binding affinity observed at arginine 123 (Arg123) (ΔEtotal = −167.90 kcal/mol). PlayMolecule combined with SailVian predicted three potential binding sites for Lumefantrine on FLI-1 (Figures 3D–F). The optimal docking pose exhibited a binding score of −7.9, comparable to positive control molecules TK216 (−7.9) and YK-4-279 (−7.6).

Molecular dynamics simulations revealed stable complex formation (Figure 3G): Root mean square deviation (RMSD) stabilized at 2.0–2.2 nm after 20 ns equilibration (Figure 3H). Root mean square fluctuation (RMSF) analysis indicates residue 20-60/220-240 flexibility, while active site conformation preserved (Figure 3I). The radius of gyration (Rg) of the protein stabilized between 2.4–2.8 nm, reflecting stable folding influenced by secondary structure (Figure 3J). Decreased solvent-accessible surface area (SASA) indicates a tightly packed, stable complex (Figure 3K). Total binding energy was calculated to be −45.25 kcal/mol, dominated by nonpolar energy (−38.7 kcal/mol).

### 3.4 Experimental validation of FLI-1 targeting

**FIGURE 4:**
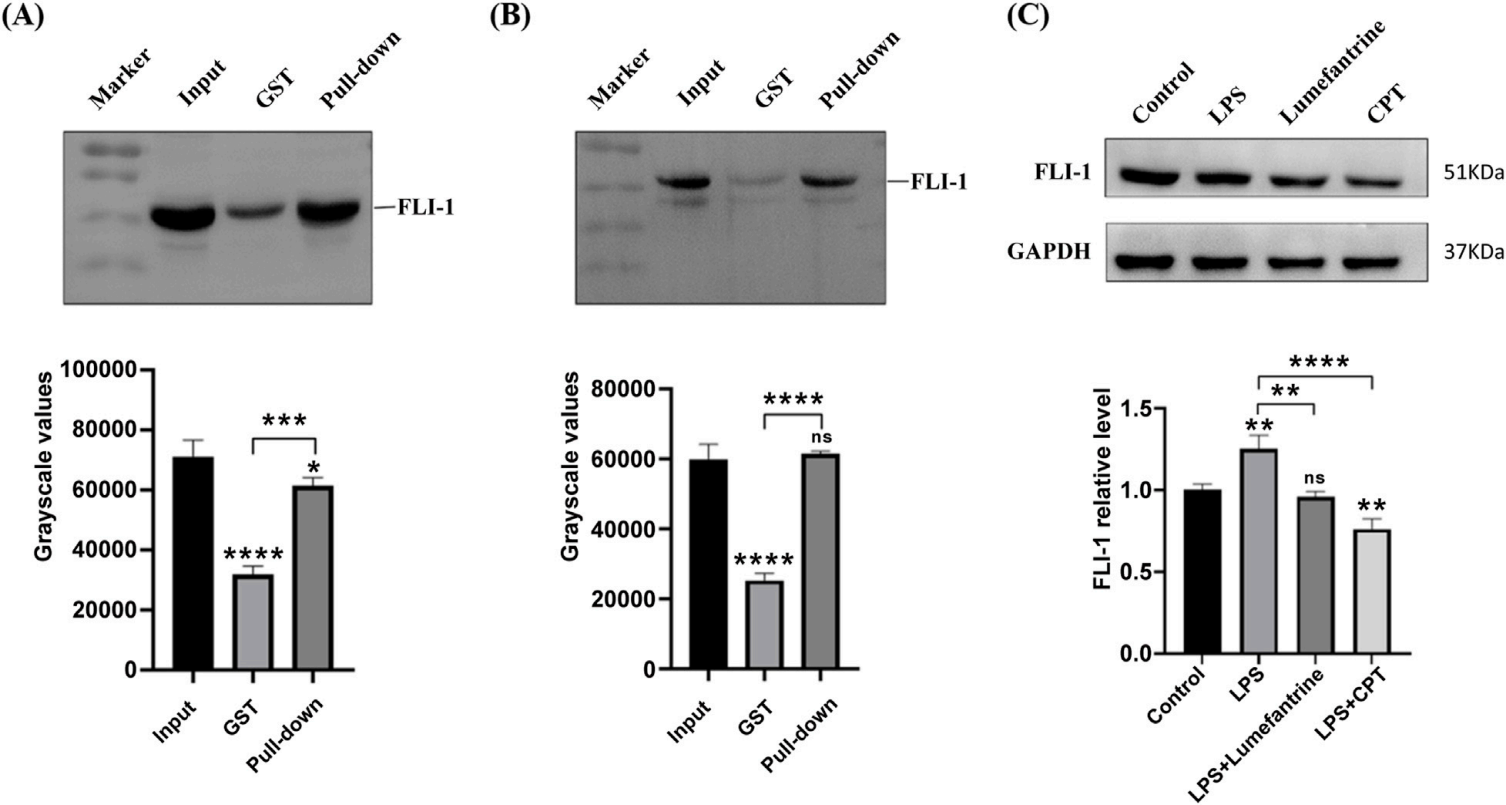
Binding of Lumefantrine to FLI-1 protein. Pull-down analysis of Lumefantrine conjugated magnetic beads and cell lysates from **(A)** IEC-6 and **(B)** NCM460, followed by WB detection using FLI-1 antibody. **(C)** Western blot was used to detect the expression of FLI-1 protein in IEC-6 after LPS modeling and treatment with Lumefantrine or Camptothecin (CPT).

To experimentally validate the Lumefantrine-FLI-1 interaction predicted by computational analyses, we performed affinity-based pull-down assays. FLI-1 was selectively enriched in Lumefantrine-conjugated μSphere bead lysates compared to GST-tagged negative controls (Figures 4A,B), confirming direct binding between Lumefantrine and FLI-1. Western blot analysis further revealed FLI-1 suppression by Lumefantrine, comparable to the inhibitory effects of Camptothecin (CPT), a known FLI-1 antagonist (Figure 4C).

### 3.5 NF-κB pathway modulation by Lumefantrine

**FIGURE 5:**
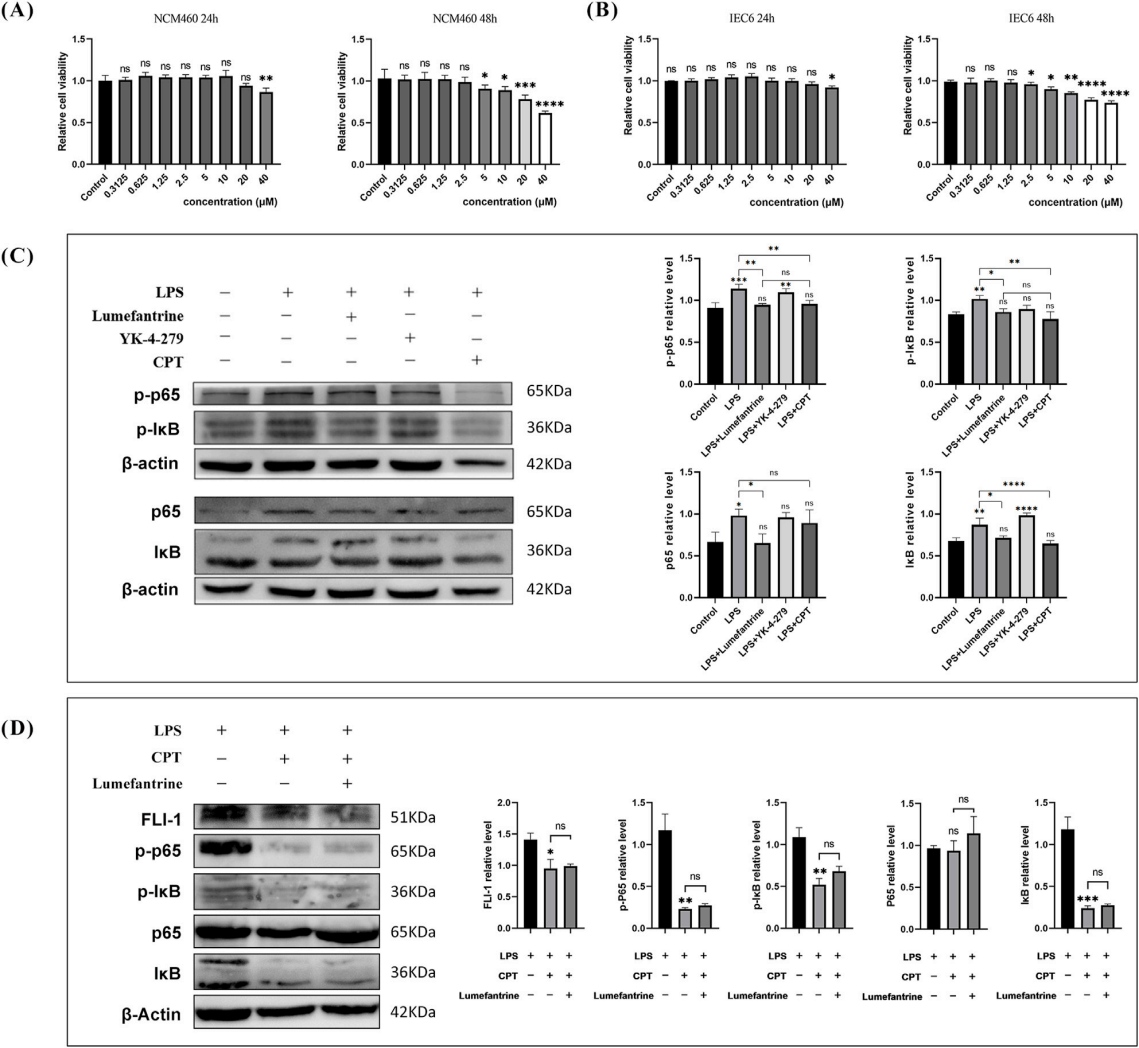
Lumefantrine modulates the NF-κB pathway. **(A,B)** CCK-8 assay showing cytotoxicity of Lumefantrine in NCM460 and IEC-6 cells after 24/48 h exposure. **(C)** Western blot analysis demonstrating Lumefantrine’s suppression of LPS-induced phosphorylation of p65 and IκB. FLI-1 inhibitors YK-4-279 and CPT used as positive controls. **(D)** Western blot assessment of Lumefantrine’s effect on NF-κB pathway proteins under CPT-mediated FLI-1 suppression.

CCK-8 assays demonstrated negligible cytotoxicity at concentrations below 40 μM during 24-h exposure in intestinal epithelial cells IEC-6 and NCM460 (Figures 5A,B). Thus, 20 μM Lumefantrine was employed for subsequent mechanistic investigations. Western blot analysis demonstrated that Lumefantrine significantly suppressed LPS-induced phosphorylation of both p65 and IκB (Figure 5C). Notably, Lumefantrine exhibited NF-κB inhibitory efficacy comparable to established FLI-1 inhibitors YK-4-279 and CPT, which served as positive controls in this study. Critically, when FLI-1 was inhibited by CPT, Lumefantrine’s suppression of p-p65 and p-IκBα was abolished (Figure 5D), confirming that its NF-κB inhibition operates specifically through FLI-1 targeting.

**FIGURE 6:**
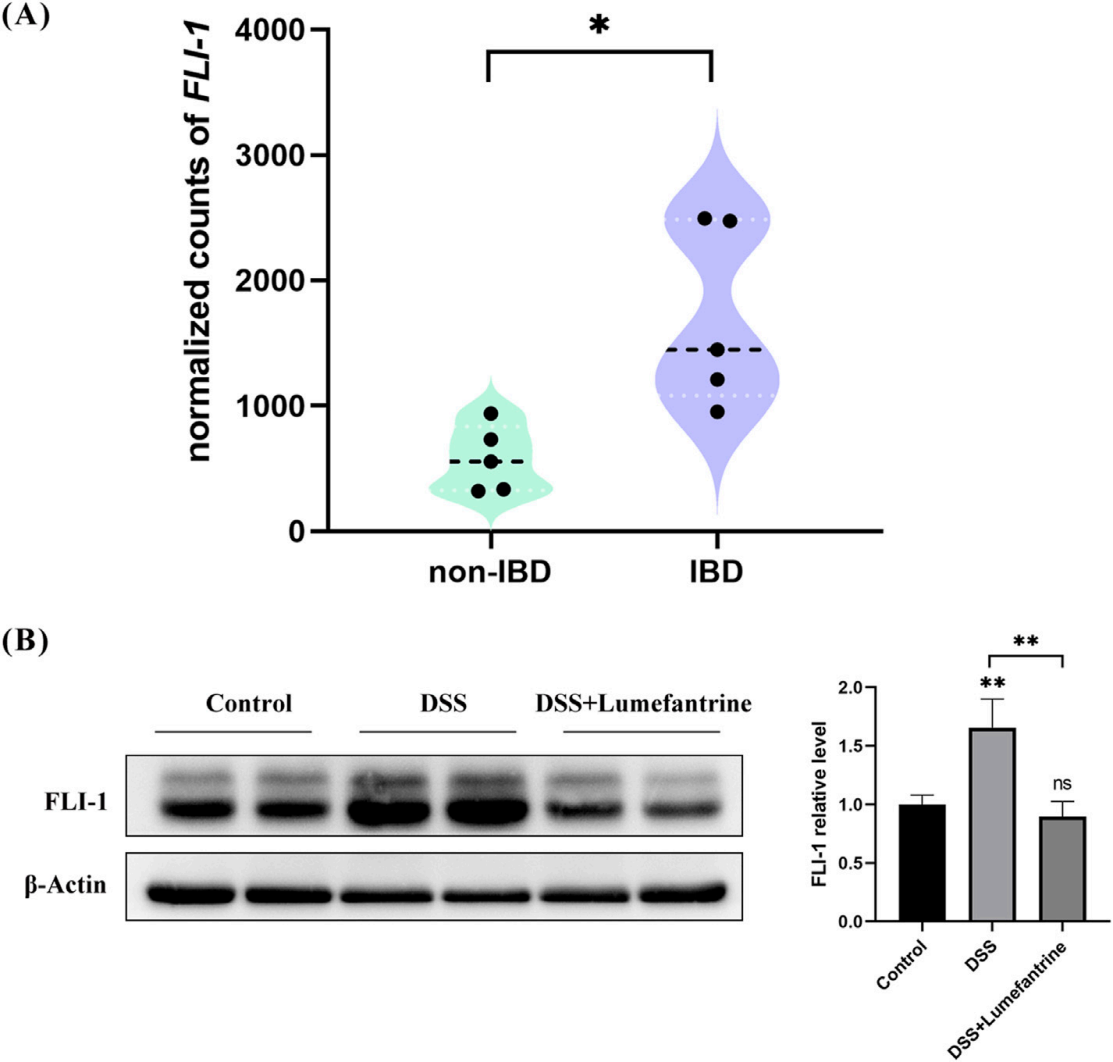
FLI-1 overexpression in IBD colonic tissues and its suppression by Lumefantrine. **(A)** Differential expression of FLI-1 in intestinal tissues between healthy individuals and IBD patients. Data sourced from the GSE255720 dataset (RNA-Seq libraries constructed from single-cell suspensions of colon biopsy tissues from 5 non-IBD healthy controls and 5 IBD patients), *p* = 0.011. **(B)** Western blot analysis of FLI-1 protein expression in normal controls, DSS-induced colitis mice and DSS mice treated with Lumefantrine.

Building upon FLI-1’s regulatory role in NF-κB signaling, we explored its therapeutic relevance in IBD. Analysis of public transcriptome datasets revealed significantly elevated FLI-1 expression in intestinal tissues from IBD patients compared to healthy controls (Figure 6A). Consistently, our DSS-induced colitis model showed increased colonic FLI-1 expression, which was effectively suppressed by Lumefantrine treatment (Figure 6B). These results establish FLI-1 as a promising therapeutic target in IBD, with Lumefantrine exerting its anti-inflammatory effects through direct FLI-1 binding and subsequent suppression of the NF-κB signaling cascade.

## 4 Discussion

Inflammatory bowel disease (IBD) remains a clinical enigma due to its complex pathogenesis, relapsing nature, and potential for neoplastic transformation (Ko and Auyeung, 2014). This chronic immune-mediated disorder is characterized by persistent mucosal inflammation, oxidative stress, and inflammatory mediator activation. Lumefantrine, a mefloquine-class antimalarial agent developed by the Chinese Academy of Military Sciences, was approved by the U.S. Food and Drug Administration (FDA) in 2009 for treating acute non-severe malaria in adults and pediatric populations (Okwu et al., 2025). Clinically employed in combination regimens to enhance therapeutic efficacy and mitigate drug resistance, Lumefantrine demonstrates a favorable safety profile with minimal adverse effects (Ippolito et al., 2017; Omari et al., 2005). Our study revealed its previously unrecognized therapeutic potential in attenuating IBD progression through novel mechanisms.

Using a dextran sulfate sodium (DSS)-induced murine colitis model that recapitulates human IBD pathology, we demonstrated Lumefantrine’s capacity to preserve intestinal mucosal architecture and suppress inflammatory cascades. Model group mice exhibited characteristic disease manifestations including progressive body weight loss, loose stools, hematochezia, and significant colon shortening, accompanied by sustained elevation of Disease Activity Index (DAI) scores–observations consistent with established IBD murine models (Wirtz et al., 2017; Mizoguchi, 2012; He et al., 2024). In contrast, Lumefantrine-treated groups showed marked symptom alleviation, with declining DAI scores observed between days 6–7 and significantly attenuated colon shortening compared to vehicle controls.

The intestinal barrier–comprising mechanical, immunological, biological, and chemical components–crucially depends on tight junction integrity as the primary defense line of mucosal protection (Dunleavy et al., 2023). Intestinal epithelial tight junctions regulate paracellular permeability and maintain epithelial homeostasis (Parikh et al., 2019). Immunohistochemical and Western blot analysis revealed Lumefantrine-mediated restoration of Claudin-1 and ZO-1 expression at colonic tight junctions, mechanistically confirming its barrier-protective effects through structural reinforcement of the mechanical barrier.

Inflammatory cytokines IL-1β, IL-6, and TNF-α drive IBD pathogenesis via various mechanisms: IL-1β compromises epithelial permeability (Bulek et al., 2020), IL-6 potentiates adaptive immune dysregulation and carcinogenesis(Hunter and Jones, 2015; Taniguchi and Karin, 2014), while TNF-α amplifies oxidative stress and chemokine production(Wang et al., 2017; Oikonomopoulos et al., 2013). Our ELISA and qRT-PCR data concordantly showed Lumefantrine’s suppression of these cytokines in serum and colon tissue, highlighting its anti-inflammatory efficacy.

Mechanistically, Lumefantrine targeted the NF-κB pathway—a master regulator of inflammatory responses. By inhibiting IκBα and p65 phosphorylation, it attenuated NF-κB nuclear translocation and suppressed transcriptional activation of *Cox-2* and *iNos*. COX-2, minimally expressed in normal tissues, becomes markedly upregulated during inflammation, driving prostaglandin E2 (PGE2) overproduction that amplifies inflammatory cascades (Singer et al., 1998). Similarly, while nitric oxide (NO) serves as a physiological signaling molecule in gastrointestinal homeostasis, pathological overexpression of iNOS drives excessive NO generation that damages intestinal epithelium and perpetuates inflammation (Cross and Wilson, 2003). Critically, these inflammatory mediators further activate NF-κB signaling through a pathogenic feedback loop, creating a self-reinforcing cycle of mucosal injury (Nie et al., 2023; Hassanein et al., 2022; Pan et al., 2011). Notably, multiple clinically approved IBD therapeutics exert their efficacy through NF-κB pathway inhibition (Atreya et al., 2008), underscoring the translational relevance of targeting this axis. Our findings position Lumefantrine as a promising candidate for modulating dysregulated NF-κB activation—a strategy with demonstrated therapeutic potential in inflammatory pathologies.

Through computational docking and experimental validation, we identified FLI-1, an ETS-family transcription factor, as the direct molecular target of Lumefantrine. This finding aligns with previous reports demonstrating that Lumefantrine inhibit FLI-1 expression to suppress glioblastoma multiforme (GBM) progression (Rajesh et al., 2020). While FLI-1 has been implicated as a therapeutic target in multiple malignancies such as Ewing sarcoma, hemangioma, and squamous cell carcinoma (Zhang et al., 2024; Yasir et al., 2023; Li et al., 2022; Ben-David et al., 2022), and autoimmune diseases such as systemic sclerosis (SSc) and systemic lupus erythematosus (sLE) (He et al., 2021), our work newly positions FLI-1 as a potential therapeutic target for IBD. Public transcriptomic datasets revealed elevated FLI-1 expression in IBD lesions compared to healthy intestinal tissues. Consistent with this observation, FLI-1 upregulation was recapitulated in our DSS-induced murine colitis model. FLI-1 inhibition by Lumefantrine disrupts its regulatory role in NF-κB activation, leading to reduced phosphorylation of p65 and IκBα. This aligns with prior findings in diffuse large B-cell lymphoma models, where FLI-1 knockdown attenuated NF-κB1 (p50) and RelA (p65) expression (Sartori et al., 2021). Notably, Lumefantrine’s NF-κB inhibitory efficacy mirrored that of established FLI-1 inhibitors–Camptothecin (Wang et al., 2023) and YK-4-279 (Huang et al., 2021). Critically, under CPT-mediated FLI-1 suppression, Lumefantrine failed to modulate the expression of NF-κB signaling components p-p65 and p-IκB. Collectively, Beyond validating Lumefantrine’s therapeutic potential for IBD, our findings suggest FLI-1 as a novel and actionable target warranting further exploration.

## Data Availability

The original contributions presented in the study are included in the article/Supplementary Material, further inquiries can be directed to the corresponding author.
