# Supplementary material for: Lumefantrine ameliorates DSS-induced colitis by targeting FLI-1 to suppress NF-κB signaling
Source: Front Pharmacol. 2025 Jul 11;16:1614978. doi: 10.3389/fphar.2025.1614978 (PMC12289662; doi:10.3389/fphar.2025.1614978)
Supplement: Supplementary file 1 [file Table1.docx]

Supplementary Material

# Table 1. DAI Scoring Criteria

| **Weight Loss (%)** | **Hematochezia** | **Stool Consistency** | **Score** |
| --- | --- | --- | --- |
| 0 | Negative | Normal | 0 |
| 1-5 | Positive | Soft | 1 |
| 5-10 | Positive | Very soft | 2 |
| 10-15 | Positive | Loose | 3 |
| >15 | Gross bleeding | Diarrhea | 4 |

# Table 2. Histopathological Scoring Criteria

| **Lesion Depth** | **Crypt Damage** | **Inflammatory Infiltration** | **Score** |
| --- | --- | --- | --- |
| None | None | None | 0 |
| Mucosal layer | Basal 1/3 | Mild | 1 |
| Submucosa | Basal 2/3 | Moderate | 2 |
| Muscularis propria | Surface epithelium only | Severe | 3 |
| Transmural | Complete crypt loss | Severe | 4 |

# Table 3. Primer Sequences

| Gene Name | Primer sequence |
| --- | --- |
| *IL-1β* | Forward: CCAACAAGTGATATTCTCCATG |
|  | Reverse: ATTGCTTGGGATCCACACTCT |
| *IL-6* | Forward: GAGAAAAGAGTTGTGCAATGG |
|  | Reverse: GTACTCCAGGTAGCTATGGTA |
| *Tnf-α* | Forward: CTCACACTCAGATCATCTTCTC |
|  | Reverse: CCTGGGAGTAGACAAGGTACA |
| *Cox-2* | Forward: TGGTCTGGTGCCTGGTCTGATG |
|  | Reverse: GCGGTTCTGATACTGGAACTGCTG |
| *iNos* | Forward: GATGTGCTGCCTCTGGTCTTGC |
|  | Reverse: CAGCCACATTGATCTCCGTGACAG |
| *β-Actin* | Forward: TGGGTCAGAAGGACTCCTATG |
|  | Reverse: GTAGAAGGTGTGGTGCCAGAT |
